# Comparison of the utility and validity of three scoring tools to detect skin disease in patients with juvenile dermatomyositis

**DOI:** 10.1186/1546-0096-12-S1-P93

**Published:** 2014-09-17

**Authors:** Raquel Campanilho-Marques, Beverley Almeida, Katie Arnold, Kiran Nistala, Clarissa A Pilkington, Lucy R Wedderburn

**Affiliations:** 1 Infection, Inflammation and Rheumatology Section, UCL ICH, London, United Kingdom; 2 Department of Rheumatology, Great Ormond Street Hospital for Children NHS Trust, London, United Kingdom; 3 Centre for Rheumatology, UCL, London, United Kingdom; 4 Arthritis Research UK Centre for Adolescent Rheumatology, UCL, UCLH and GOSH, London, United Kingdom

## Introduction

Juvenile dermatomyositis (JDM) is a rare condition affecting 3 children/million/year. Muscle and skin involvement are key features. The muscle symptoms are frequently the main initial focus: formal measures (CMAS and MMT8) exist to routinely and accurately assess this component of the disease. However the involvement of skin, and its assessment, is also a vital aspect. The abbreviated Cutaneous Assessment Tool (CAT) encompassing active skin disease and skin damage, Disease Activity Score (DAS) and Myositis Intention to Treat Activity Index (MITAX), both with skin components, have all been suggested to measure skin disease in JDM; however the optimal tool is unknown.

## Objectives

To compare three tools for assessment of skin disease in JDM and correlate them with the physician’s 10cm skin visual analogue scale (physician’s skin VAS) to define which tool best assesses skin disease.

## Methods

Patients recruited to the UK JDM Cohort & Biomarker Study who fulfil Bohan-Peter criteria for JDM were included. Each patient was assessed for skin disease using the CAT, DAS, MITAX and an overall physician’s skin VAS. Markers of muscle disease (CMAS, MMT8, CK U/L), inflammatory markers (CRP mg/L and ESR mm/hr) and overall physician’s global score were also recorded. Spearman’s correlations (r_s_) were used to correlate categorical and continuous variables and a relationship >0.75 was considered strong. A p-value <0.05 was considered significant.

## Results

Between 2012 and 2014, 67 JDM patients were assessed. 59.7% were female. The mean (±SD) age of the patients was 9.86 ±3.37 years, with mean age at diagnosis 6.59 ±3.42 years and mean disease duration of 3.26 ±3.08 years. The skin section of the DAS had the strongest correlation with the physician’s skin VAS (Table 1). The skin section of the MITAX and the CAT activity scores were significantly correlated with the physician’s skin VAS. DAS skin, MITAX skin and CAT Activity scores were all negatively correlated with CMAS and MMT8 scores; no significant correlations were noted with the CK. DAS skin scores were significantly correlated with both the CRP and ESR, while the MITAX skin was significantly correlated only with the ESR, and CAT Activity only with the CRP.

**Table 1:** Spearman’s correlation between items shown as r_s_ and corresponding p value

	Physician’s skin VAS n=67	**CMASn=67**	**MMT8n=67**	**CKn=52**	**CRPn=55**	**ESRn=54**
**DAS skin**	r_s_ 0.795 p<0.001	r_s_ -0.443 p<0.001	r_s_ -0.424 p<0.001	r_s_ 0.176 p 0.212	r_s_ 0.280 p 0.039	r_s_ 0.311 p 0.022

**MITAX skin**	r_s_ 0.594 p<0.001	r_s_ -0.404 p 0.001	r_s_ -0.453 p<0.001	r_s_ 0.177 p 0.210	r_s_ 0.208 p 0.127	r_s_ 0.281 p 0.040

**CAT Activity**	r_s_ 0.623 p<0.001	r_s_ -0.471 p<0.001	r_s_ -0.428 p<0.001	r_s_ 0.157 p 0.267	r_s_ 0.300 p 0.026	r_s_ 0.164 p 0.235

## Conclusion

These data demonstrate the potential application of using a skin assessment tool to evaluate and monitor skin involvement in JDM patients. It also demonstrates that the DAS skin section appears to be the best of the tools using the physician’s skin VAS as the gold standard. The DAS skin tool was concise, quick to use and easy to score.

## Disclosure of interest

None declared.

